# Hippocampal theta oscillations synchronize with rhythmical head movements during locomotion

**DOI:** 10.1186/1471-2202-12-S1-P266

**Published:** 2011-07-18

**Authors:** Anders Ledberg, David Robbe

**Affiliations:** 1 Center for Brain and Cognition, Dept of Information and Communication Technologies, Universitat Pompeu Fabra, Barcelona, Spain; 2 Institut d’Investigacions Biomèdiques August (IDIBAPS), Barcelona, Spain

## 

The hippocampal theta rhythm is required for accurate navigation and spatial memory but its relation to sensorimotor activities associated with locomotion are poorly understood. We addressed this issue by precisely quantifying the rhythmical dynamics of locomotion while simultaneously recording hippocampal neuronal activity in freely moving rats. We report that when rats run, their heads oscillate at 6-12 Hz in a speed-dependent manner. This periodic movement modulates the phases and amplitudes of the hippocampal theta rhythm, suggesting a mechanism to update the hippocampal network activity with information about self-movement.

Three rats were trained to collect drops of water alternatively delivered at the two extremities of a 1.6 meter long maze. Hippocampal local field potentials (LFPs) were recorded close to the pyramidal layer in the dorsal part of hippocampal area CA1. We quantified the rhythmical dynamics of locomotion by recording vertical head acceleration using a miniature accelerometer attached to the head of the rats.

When the rats run, the acceleration signal showed prominent oscillations along the vertical axis (up and down). The peak frequency of these oscillations was close to 9 Hz, very similar to the frequency of the simultaneously recorded hippocampal theta. We estimated the 'instantaneous' frequency, phase and amplitude of the LFP and vertical head oscillations by finding the sine wave that best described the data in short sliding windows. Phase synchrony was investigated by computing the difference of the estimated phases for pairs of data windows in which both LFP and head acceleration show strong power in the theta band and moreover had similar frequencies. The phase differences for the three rats deviated significantly from a uniform distribution. Given the sinusoidal nature of the oscillations, this is a strong indication of phase synchrony. Further evidence for a modulation of hippocampal theta by the movement dynamics was provided by the observation that theta amplitude was highest when hippocampal theta and head oscillations were in phase.

To better understand the mechanism behind these hippocampal theta modulations we constructed a simple model of hippocampal theta activity. When this model is forced by an input proportional to the head acceleration signal, it shows phase-locking and amplitude-modulation quantitatively in agreement with the experimental results. This suggests that the hippocampus receives inputs phase-locked to the head movements, e.g. through the vestibular system, and that these inputs effectively forces the theta rhythm to synchronize to the dynamics of locomotion.

